# Supplementary material for: Patterns of Intron Gain and Loss in Fungi
Source: PLoS Biol. 2004 Nov 30;2(12):e422. doi: 10.1371/journal.pbio.0020422 (PMC532390; doi:10.1371/journal.pbio.0020422)
Supplement: Table S1 — Also available at http://genes.mit.edu/NielsenEtAl/. (4.3 MB ZIP). [file pbio.0020422.st001.zip › NielsenEtAl/html/238.html]

AN2981.1.NCU09111.1.MG09926.2.FG01279.1


```
 CLUSTAL W (1.82) Multiple Sequence Alignments - Introns Inserted


Sequence 1: MG09926.2	501 aa
Sequence 2: FG01279.1	497 aa
Sequence 3: NCU09111.1	491 aa
Sequence 4: AN2981.1	511 aa
Alignment Length: 513 aa
Number Identitical Residues: 394 aa
Alignment Score (without introns) 16650


MG09926.2 	------------MDS~ELE~LKDNTVIVVLGASGDLAKKKT0YPALFGL0YRNQFLPKDI
NCU09111.1	---------------~MEQ1LKDNTVIVVLGASGDLAKKKT0Y------~-RNQFLPKDI
FG01279.1 	---------------~-ME~LKQNTTIVVLGASGDLAKKKT0YPALFGL0YRNQFLPKDV
AN2981.1  	MSATIARAEEQQNGS2TVE~LKDDTVIVVLGASGDLAKKKT0FPALFGL0FRNKFLPKGI
          	 ::: : :.... .:   : **::*.*************** :.:  .   **:****.:

MG09926.2 	KIVGYARTKMDHEEYIRRIRSYIKTPTKDMEQQLDDFCSLCTYVAGQYDKDESFYNLNDH
NCU09111.1	RIVGYARTKMDHDEYIRRIKSYIKTPTKESEQQLEEFCSICTYVSGQYDRDDSFLQLNKH
FG01279.1 	KIVGYARTKMDHDEYIRRIKSYIKTPTKEIEQQLEDFAALCTYVSGQYDKDESFQGLEQH
AN2981.1  	KIVGYARTQMDHNEYLKRVRSYIKTPTKEIEEQLNSFCELCTYISGQYDQDDSFKNLAKH
          	:*******:***:**::*::********: *:**:.*. :***::****:*:**  * .*

MG09926.2 	LEDLEKNQSEAHRLFYMALPPSVFTIVSQHLKRCCYPTRGIARVI0VEKPFGKDLASSRE
NCU09111.1	LEELEQGRKENNRLFYMALPPSVFTIVSQHLKKCCYPSRGVARVI0VEKPFGKDLASSRE
FG01279.1 	LQEVEASRPENHRLFYMALPPSVFTIVSQHLKKICYPKNGIARVI0VEKPFGKDLASSRE
AN2981.1  	LEEIEKNQKEQNRVFYMALPPSVFITVSEQLKRNCYPKNGVARII0VEKPFGKDLQSSRD
          	*:::* .: * :*:**********  **::**: ***..*:**:* ********* ***:

MG09926.2 	LQKSLEPDWKEDELFRIDHYLGKEMVKNILILRFGNSFLGATWNRHHIDNVQ~ITFKEPF
NCU09111.1	LQKSLEPDWKEEEIFRIDHYLGKEMVKNILILRFGNSFLGATWNRHHIDNVQ~ITFKEPF
FG01279.1 	LQKSLEPDWNEQELFRIDHYLGKEMVKNILILRFGNSFLGATWNRHHIDNVQ~ITFKEPF
AN2981.1  	LQKALEPNWKEEEIFRIDHYLGKEMVKNILIMRFGNEFFNATWNRHHIDNVQ0ITFKEPF
          	***:***:*:*:*:*****************:****.*:.************ *******

MG09926.2 	GTEGRGGYFDEFGIIRDVMQNH~LLQVLTLLAMERPISFSSEDIRDEK~VRVLRAIPAIE
NCU09111.1	GTEGRGGYFDEFGIIRDVMQNH~LLQVLTLLAMERPISFSAEDIRDEK~VRVLRAIPAIE
FG01279.1 	GTEGRGGYFDEFGIVRDVMQNH~LLQVLTLLAMERPISFNAEDIRDEK~VRVLRAIPAIE
AN2981.1  	GTEGRGGYFDEFGIIRDVMQNH1LLQVLTLLAMERPISFSAEDIRDEK0VRVLRAMDPIQ
          	**************:******* ****************.:******* ******: .*:

MG09926.2 	PKNVIIGQYGRSLDGSKPSYKEDDTVPKESRCPTFCALVAYIKNERWDGVPFIMKAGKA~
NCU09111.1	PKNVIIGQYGKSLDGSKPAYKEDDTVPKDSRCPTFCALVAYIKNERWDGVPFIMKAGKA~
FG01279.1 	PKNVIIGQYGKSLDGSKPAYREDDTVPQDSRCPTFCALVAYIKNERWDGVPFIMKAGKA~
AN2981.1  	PKDVIIGQYGRSLDGSKPAYKEDDTVPQDSRCPTFCALVAHIKNERWDGVPFIMKAGKA1
          	**:*******:*******:*:******::***********:****************** 

MG09926.2 	LNEQKTEIRIQFKDVTSGIFKDIPRNELVMRIQPNESVYIKMNSKLPGLSMQTVVTELDL
NCU09111.1	LNEQKTEIRVQFKDVTSGIFKDIPRNELVMRIQPNESVYIKMNSKLPGLSMQTVVTELDL
FG01279.1 	LNEQKTEIRIQFKDVTSGIFKDIPRNELVMRIQPNESVYIKMNSKLPGLSMQTVVTELDL
AN2981.1  	LNEQKTEIRIQFKDVTSGIFKDIPRNELVIRVQPNESVYIKMNSKLPGLSMQTVVTELDL
          	*********:*******************:*:****************************

MG09926.2 	TYRRRFSDLKIPEAYESLILDCLKGDHSNFVRDDELDASWRIFTPLLHYLDDNKEIVPME
NCU09111.1	TYRRRFSDLKIPEAYESLILDCLKGDHSNFVRDDELDASWRIFTPLLHYLDDNKEIIPME
FG01279.1 	TYRRRFSDLKIPEAYESLVLDCLKGDQSNFVRDDELDASWRIFTPLLHYLDDNKEIIPME
AN2981.1  	TYRRRFSDLKIPEAYESLILDALKGDHSNFVRDDELDASWRMFTPLLHYLDDNKEIIPME
          	******************:**.****:**************:**************:***

MG09926.2 	YPYG1SRGPAVLDDFTASYGYKFSDAAGYQ2WPTTSAALHANKL
NCU09111.1	YPYG1SRGPAVLDDFTASYGYKFSDAAGYQ~WPTTSAMGSGMKL
FG01279.1 	YPYG1SRGPAVLDDFTSSYGYKFSDAAGYQ~WPTTNAAAAPNKL
AN2981.1  	YPYG1SRGPSVLDDFTASYGYKFSDAAGYQ~WPLTHTT--PNRL
          	**** ****:******:************* ** * :     :*
```
